# UBE3C restricts EV-A71 replication by ubiquitination-dependent degradation of 2C

**DOI:** 10.1128/jvi.01335-24

**Published:** 2024-08-30

**Authors:** Boming Cui, Ge Yang, Haiyan Yan, Shuo Wu, Kun Wang, Huiqiang Wang, Yuhuan Li

**Affiliations:** 1 CAMS Key Laboratory of Antiviral Drug Research, Beijing Key Laboratory of Antimicrobial Agents, NHC Key Laboratory of Biotechnology of Antibiotics, Institute of Medicinal Biotechnology, Chinese Academy of Medical Sciences and Peking Union Medical College, Beijing, China; 2 State Key Laboratory of Bioactive Substances and Functions of Natural Medicines, Institute of Medicinal Biotechnology, Chinese Academy of Medical Sciences and Peking Union Medical College, Beijing, China; Loyola University Chicago-Health Sciences Campus, Maywood, Illinois, USA

**Keywords:** EV-A71, UBE3C, 2C, ubiquitination

## Abstract

Ubiquitin modification of viral proteins to degrade or regulate their function is one of the strategies of the host to resist viral infection. Here, we report that ubiquitin protein ligase E3C (UBE3C), an E3 ubiquitin ligase, displayed inhibitory effects on EV-A71 replication. UBE3C knockdown resulted in increased viral protein levels and virus titers, whereas overexpression of UBE3C reduced EV-A71 replication. To explore the mechanism by which UBE3C affected EV-A71 infection, we found that the C-terminal of UBE3C bound to 2C protein and facilitated K33/K48-linked ubiquitination degradation of 2C K268. Moreover, UBE3C lost its ability to degrade 2C K268R and had a diminished inhibitory impact against the replication of recombinant EV-A71-FY-2C K268R. In addition, UBE3C also promoted ubiquitination degradation of the 2C protein of CVB3 and CVA16 and inhibited viral replication. Thus, our findings reveal a novel mechanism that UBE3C acts as an enterovirus host restriction factor, including EV-A71, by targeting the 2C protein.

**IMPORTANCE:**

The highly conserved 2C protein of EV-A71 is a multifunctional protein and plays a key role in the replication cycle. In this study, we demonstrated for the first time that UBE3C promoted the degradation of 2C K268 *via* K33/K48-linked ubiquitination, thereby inhibiting viral proliferation. Our findings advance the knowledge related to the roles of 2C in EV-A71 virulence and the ubiquitination pathway in the host restriction of EV-A71 infection.

## INTRODUCTION

Enterovirus A71 (EV-A71), belonging to the genus *Enterovirus* of the *Picornaviridae* family, is a prominent pathogen that causes hand, foot, and mouth disease (HFMD) in young kids and infants, posing a significant danger to worldwide public health (1). EV-A71 genome encoding 2,193 amino acids is translated into a polyprotein, processed into four structural proteins (VP1, VP2, VP3, and VP4), and seven nonstructural proteins (2A, 2B, 2C, 3A, 3B, 3C, and 3D). Among structural proteins and non-structural proteins, the 2C protein is highly conserved and plays various functions in the viral life cycle (2). The 2C protein consists of an N-terminal membrane-binding domain, a central ATPase domain, a cysteine-rich domain, and a C-terminal helical domain (3). Modification of amino acids in the C-terminal domain may disrupt ATPase activity and homo-oligomerization of EV-A71 2C helicase, thereby impeding virus infection (2, 4, 5).

Protein ubiquitination is a post-translational modification that works by tagging the 76 amino acid ubiquitin to the lysine residues in target proteins. Ubiquitination is catalyzed by three enzymes, including the E1 ubiquitin-activating enzyme, the E2 ubiquitin-conjugating enzyme, and the E3 ubiquitin-ligase (6). Polyubiquitin chains form through the attachment of seven lysine residues (K6, K11, K27, K29, K33, K48, and K63) or the N-terminal methionine (M1), with all ubiquitin moieties linked through the same lysine or methionine residue (7). Heterotypic chains have multiple linkage types and are further categorized into linear and branched chains. Given that K48 linkages are the most common of all ubiquitin chains, accounting for about 50% of all connections (8), this type of chain is likely to conjugate various biological proteins. The Ub-clipping method has advanced detection and quantification of branched polyubiquitin chains, revealing 10%–20% of cellular chains as branched, but few types have been identified and characterized (9). The K33/K48-branched chain was identified to have dual functions, with K11/K48- and K29/48-branched chains promoting degradation in proteasomes (10). K33/K48-linked chains act as both nondegradable and degradable signals, promoting and regulating SERINC5 expression on the cell surface. Ubiquitination and ubiquitin-like modifications also play an important role in host regulation of the EV-A71 infection (11). Our previous study showed that the E3 ligase XIAP promotes EV-A71 VP2 ubiquitin-like neddylation to reduce its stability and inhibit the replication of EV-A71 (12). Host E3 ubiquitin ligase SPOP mediates the ubiquitination and degradation of the 2A protein of EV-A71, thereby limiting EV-A71 replication (13). Therefore, gaining a deeper comprehension of the biology of ubiquitin-linked chains will provide novel insights into the overall mechanism of EV-A71 infection.

Ubiquitin protein ligase E3C (UBE3C), also known as RTA-associated ubiquitin ligase (RAUL), is a member of the HECT E3 ligase subfamily with an IQ motif at the N-terminal and an HECT domain at the C-terminal (14). Previous studies have demonstrated that UBE3C assembles the K29/K48 heterotypic ubiquitin chains, which serve as markers for proteasomal degradation (15). In excess of ubiquitinated proteins, UBE3C localizes to the proteasome and enhances proteasome processivity (16). Recent research has shown that UBE3C and TRABID regulate K29/K48-branched ubiquitination of VPS34, enhancing binding to proteasomes for degradation and suppressing autophagosome formation and maturation (10). However, another study showed that UBE3C assembled the K33-branched ubiquitin chain to ATG4B, thereby down-regulating ATG4B activity and association with LC3 to regulate autophagy under normal and starvation conditions, but did not degrade ATG4B proteins (17). Kaposi’s sarcoma-associated herpes virus immediate-early lytic cycle trigger protein RTA recruited UBE3C to directly catalyze lysine 48-linked polyubiquitination degradation of both interferon regulatory factor 7 (IRF7) and IRF3 to augment its countermeasures against the host antiviral response (18). All these findings suggest that UBE3C-mediated ubiquitin-dependent regulation serves special functions in different conditions. However, the role of UBE3C in different viral infections is still poorly understood, and more research is needed to discover its role and mechanism in viral replication.

In this study, we identified the interaction between UBE3C and the EV-A71 2C protein by combining immunoprecipitation and mass spectrometry. UBE3C binds to the C-terminal of 2C and facilitates K33/K48-linked ubiquitination of 2C K268 for degradation. We provide evidence that UBE3C-mediated 2C ubiquitination negatively regulates EV-A71 infection.

## RESULTS

### Identify the host E3 ligase that interacts with 2C

**Fig 1:**
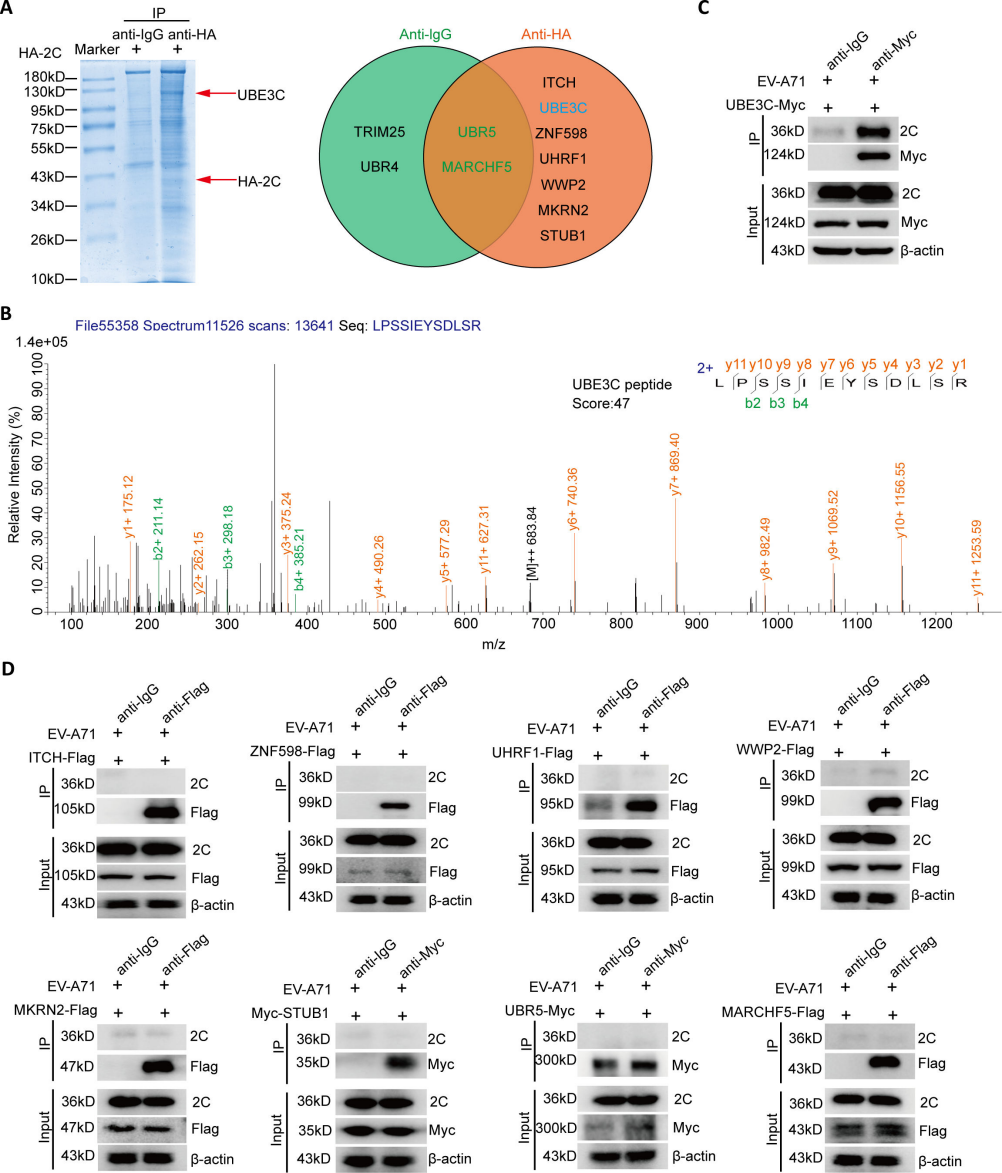
Identify the host E3 ligase that interacts with 2C. (**A,B**) The immunoprecipitates of HEK293T cells overexpressing 2C-HA were detected by SDS-PAGE Coomassie blue for LC-MS/MS assay. The 2C-HA interactome (in the red circle) was identified and compared to the normal IgG interactome (in the green circle). (**C,D**) Detection of interaction between 2C and different E3 ubiquitin ligases. HEK293T cells were transfected with different E3 ubiquitin ligase expression plasmids for 24 h. Cells were subsequently infected with EV-A71 [H, multiplicity of infection (MOI) = 0.01] for 24 h and collected for Co-IP analysis with the indicated antibodies.

To identify specific E3 ubiquitin ligases that interact with the EV-A71 2C protein in host cells, the HA-labeled EV-A71 2C protein was expressed in HEK293T cells and subjected to anti-HA antibody immunoprecipitation and Coomassie blue staining (Fig. 1A). The proteins in the pull-down protein bands were analyzed by liquid chromatography with tandem mass spectrometry (LC-MS/MS), and the results showed that the anti-IgG group predicted 1,319 proteins, while the anti-2C-HA group predicted 1,766 proteins. Moreover, our results predict two host proteins COPB2 and EXPORTIN-2 that have been proven to interact with 2C (19), indicating that our immunoprecipitation system works well. Since the purpose of our study was to find E3 ligases that regulate 2C proteins, we only show the predicted E3 ligase results in Fig. 1A. Of the nine host E3 ubiquitin ligases identified by LC-MS/MS, seven E3 ubiquitin ligases were predicted to be associated with 2C after excluding non-specific binding (Fig. 1A and B). To verify the predicted interaction between E3 ubiquitin ligases and 2C protein, the above-mentioned E3 ubiquitin ligase expression plasmids were transfected into HEK293T cells for 24 h, and then cells were infected with EV-A71 for co-immunoprecipitation (Co-IP) analysis. The results showed that only UBE3C interacts with 2C protein, while the other E3 ubiquitin ligases do not interact with 2C protein (Fig. 1C and D).

### The C-terminal of UBE3C binds to 2C

**Fig 2:**
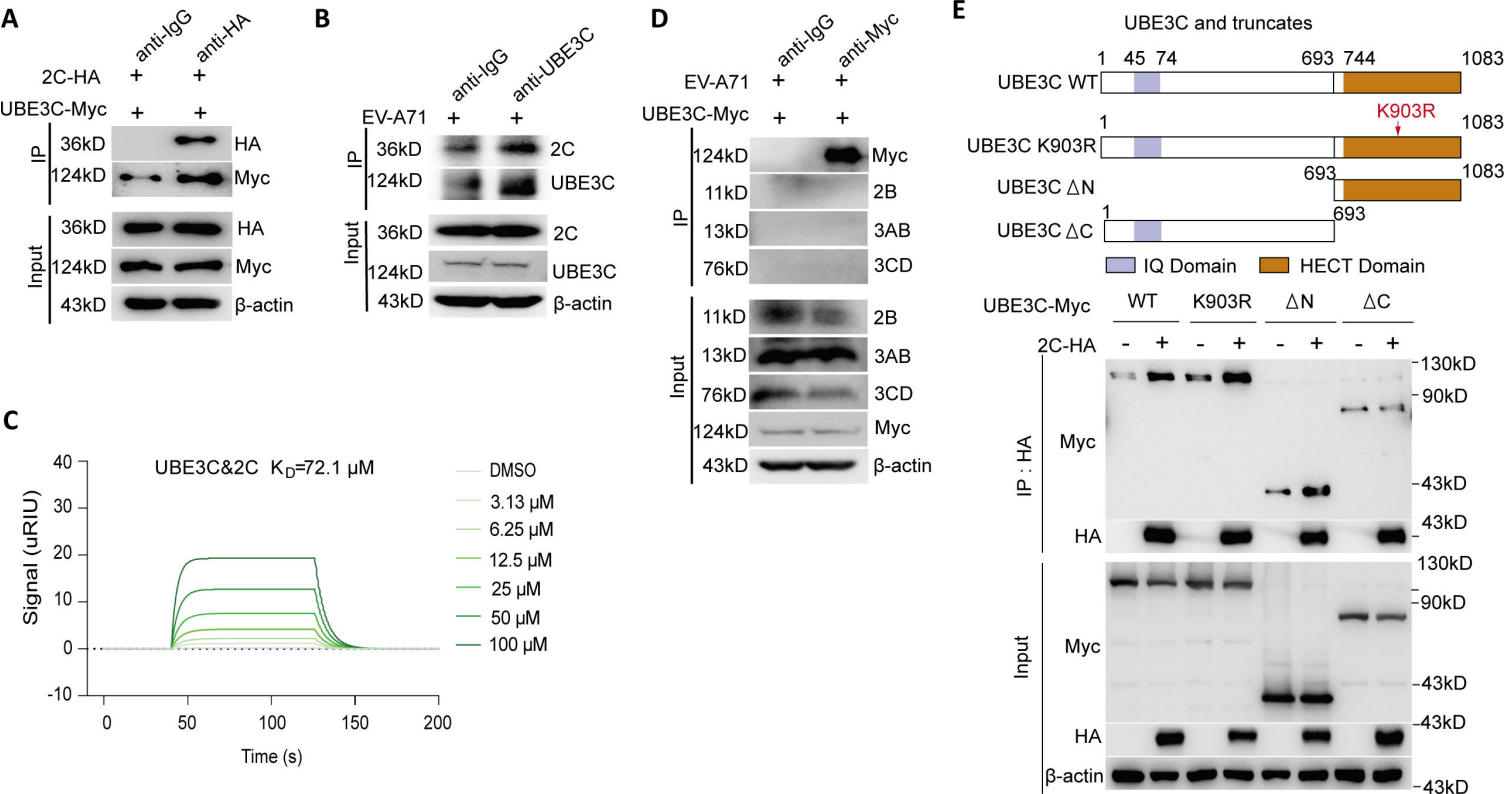
The C-terminal of UBE3C binds to 2C. (**A**) HEK293T cells were co-transfected with a plasmid expressing 2C-HA and a plasmid-expressing UBE3C-Myc. Immunoprecipitation from cell lysates was performed and probed with the indicated antibodies. (**B**) HCT-8 cells were infected with EV-A71 [H, multiplicity of infection (MOI) = 0.01] for 24 h. Immunoprecipitation from cell lysates was performed and probed with the indicated antibodies. (**C**) The *in vitro* binding of purified UBE3C to EV-A71 2C was determined by SPR binding assay. (**D**) HEK293T cells were transfected with UBE3C-Myc for 24 h, cells were subsequently infected with EV-A71 (H, MOI = 0.01) for 24 h and collected for Co-IP analysis with the indicated antibodies. (**E**) HEK293T cells were co-transfected with 2C-HA and indicated plasmids for 24 h. Immunoprecipitation from cell lysates was performed and probed with the indicated antibodies.

To further determine the interaction between 2C and UBE3C, HEK293T cells were co-transfected with UBE3C-Myc and 2C-HA plasmids for 24 h, and cells were collected for Co-IP analysis. As shown in Fig. 2A, UBE3C-Myc could also be pulled down by 2C-HA. In addition, HCT-8 cells were infected with EV-A71 for 24 h, and the cell lysates were harvested for immunoprecipitation with UBE3C antibody or an unlabeled nonspecific mouse IgG (normal IgG). As shown in Fig. 2B, an antibody against UBE3C specifically pulled down 2C, indicating that 2C interacts with endogenous UBE3C. The findings of the SPR binding experiment under *in vitro* conditions provide additional evidence of direct interaction between UBE3C and 2C (Fig. 2C). Moreover, the results showed that there was no relationship between UBE3C and non-structural proteins 2B, 3AB, and 3CD (Fig. 2D).

The UBE3C protein consists of an IQ motif at the N-terminus and a HECT domain at the C-terminus (20). Notably, Lys903 was identified as the major site of self-ubiquitination and the active site of the enzyme in the UBE3C HECT domain (20). To validate the specific domain of UBE3C that interacts with 2C, we constructed two UBE3C truncation mutants and one enzyme-inactivated mutant (K903R) and conducted co-immunoprecipitation tests. The results showed that the interaction between UBE3C and 2C was mediated by the C-terminal domain of UBE3C (Fig. 2E) and was independent of the key enzyme active site of UBE3C K903 (Fig. 2E). Overall, our results suggest that the UBE3C HECT domain has an interaction with 2C protein.

### UBE3C restricts the replication of EV-A71 *in vitro*

**Fig 3:**
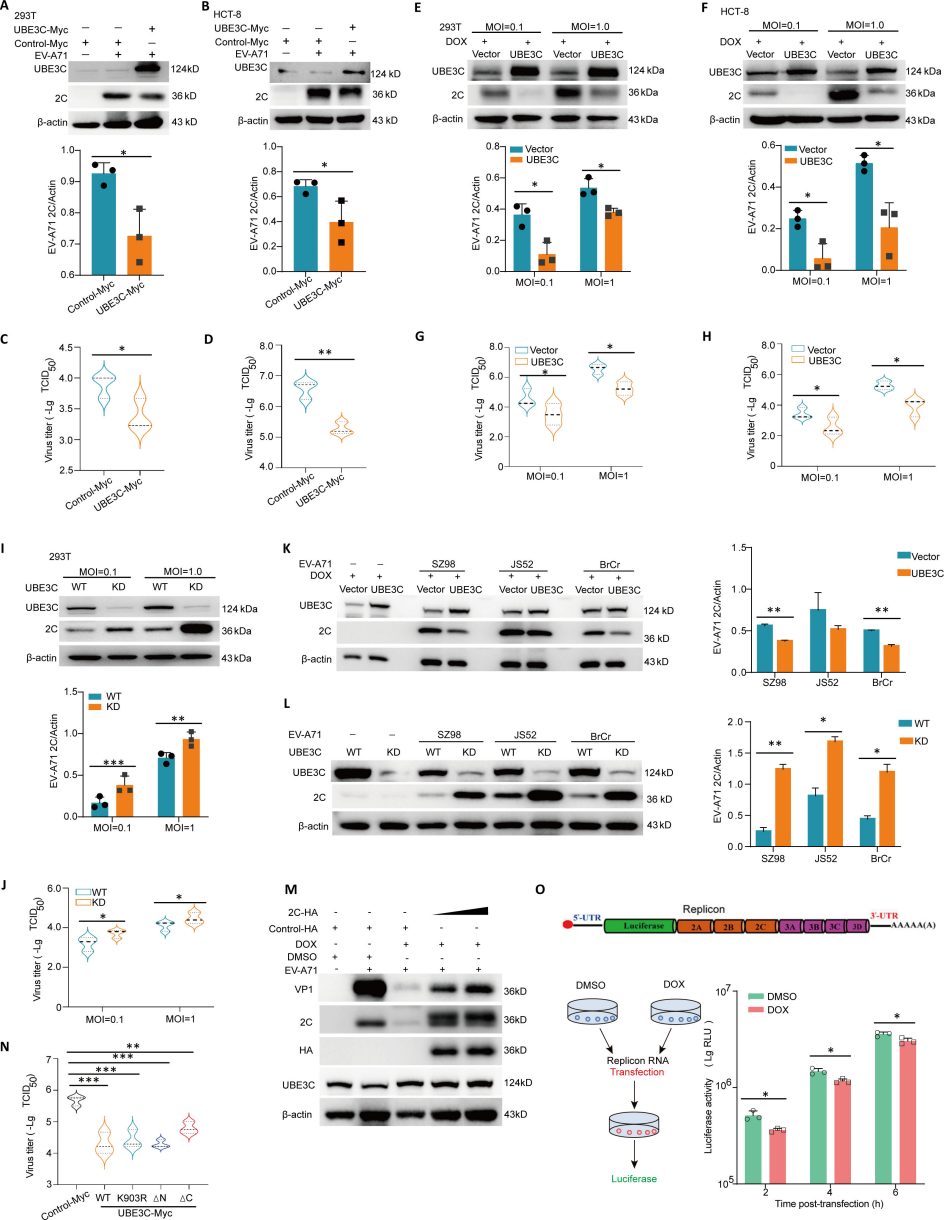
UBE3C restricts the replication of EV-A71 *in vitro*. (**A–D**) HEK293T cells or HCT-8 cells were transfected with UBE3C-Myc or control Myc plasmid for 24 h and infected with EV-A71 (H, MOI = 0.1) for another 24 h. The cells were harvested for western blot assay with indicated antibodies (A,B, *n* = 3), or virus titer assay (C,D, *n* = 3) 24 h post-infection. (**E–H**) The HEK293T or HCT-8 cell line supporting DOX-induced UBE3C expression was treated with DOX, and then cells were infected with EV-A71 (H, MOI = 0.1 or 1.0) for 24 h. The cells were harvested for western blot assay with indicated antibodies (E,F, *n* = 3) or virus titer assay (G,H, *n* = 3) 24 h post-infection. (**I, J**) UBE3C-KD HEK293T cells and wide-type HEK293T cells were infected with EV-A71 (H, MOI = 0.1) for 24 h. The cells were harvested for western blot assay with indicated antibodies (I, *n* = 3) or virus titer assay (J, *n* = 3). (**K**) The HEK293T cell line supporting DOX-induced UBE3C expression was treated with DOX, and then cells were infected with different EV-A71 strains (SZ98, JS52, or BrCr) for 24 h. The cells were harvested for western blot assay with indicated antibodies (*n* = 2). (**L**) UBE3C-KD HEK293T cells and wide-type HEK293T cells were infected with different EV-A71 strains (SZ98, JS52, or BrCr) for 24 h. The cells were harvested for western blot assay with indicated antibodies (*n* = 2). (**M**) The HCT-8 cell line supporting DOX-induced UBE3C expression was treated with DOX or dimethyl sulfoxide (DMSO), and then cells transfected with 2C-HA for 24 h and infected with EV-A71 (H, MOI = 0.1) for another 24 h. The cells were harvested for western blot assay with indicated antibodies (*n* = 3). (**N**) HEK293T cells were transfected with the indicated plasmids for 24 h and infected with EV-A71 (H, MOI = 0.1) for 24 h. Virus titers were determined in Vero cells by the cytopathic effect assay. (**O**) The HEK293T cell line supporting DOX-induced UBE3C expression was treated with DOX or DMSO, and then cells were transfected with EV-A71 subgenomic replicon RNA and luciferase reporter activities were determined at different time (*n* = 3). *P* < 0.05, one-way ANOVA with Holm-Sidak multiple comparisons test (**K**) or Student’s *t*-test (**A–J, L**).

To validate the regulatory impact of UBE3C on EV-A71, HEK293T cells and HCT-8 cells were transfected with UBE3C-Myc and control-Myc plasmids for 24 h. Subsequently, the cells were infected with EV-A71 [H, multiplicity of infection (MOI) = 0.1] and collected 24 h after infection. As shown in Fig. 3A through D, overexpression of UBE3C-Myc significantly decreased the levels of intracellular EV-A71 2C as well as virus yield in HEK293T cells and HCT-8 cells. Moreover, we used the DOX-on system to construct HEK293T and HCT-8 cell lines that induce UBE3C expression through doxycycline (DOX) treatment. Consistent with transient overexpression of UBE3C, viral 2C protein levels and viral titers were significantly decreased in cells treated with DOX to induce UBE3C expression (Fig. 3E through H). To further verify the regulatory effect of UBE3C knockdown on the proliferation of the EV-A71 virus, HEK293T cells with or without UBE3C knockdown were infected with EV-A71 for 24 h. The results showed that UBE3C knockdown significantly increased the levels of intracellular EV-A71 2C protein as well as virus yield (Fig. 3I and J). Moreover, results also showed that UBE3C knockdown resulted in increased 2C protein levels, whereas UBE3C overexpression decreased 2C protein levels during different EV-A71 strains replication (Fig. 3K and L).

Considering that 2C is targeted by UBE3C, we examined the effect of overexpression of 2C protein on the antiviral effect of UBE3C. As shown in Fig. 3M, the overexpression of 2C protein can reverse the antiviral effect of UBE3C, indicating that UBE3C can indeed play an antiviral role by regulating 2C protein. In addition, it is interesting to note that although the antiviral effect of UBE3C-ΔC is weaker than that of full-length UBE3C, the deletion of the C-terminal domain of UBE3C still has an antiviral effect (Fig. 3N). Finally, UBE3C-HEK293T cells treated with DOX to induce UBE3C expression were transfected with subgenomic replicon RNA (12), and luciferase reporter activities were determined at different times. The results showed that inducing UBE3C expression significantly decreased the luciferase activity, and thus replicon RNA replication (Fig. 3O). In short, our study shows that UBE3C plays an antiviral role in the viral replication phase.

### UBE3C facilitates K33/K48-linked ubiquitination degradation of 2C protein

**Fig 4:**
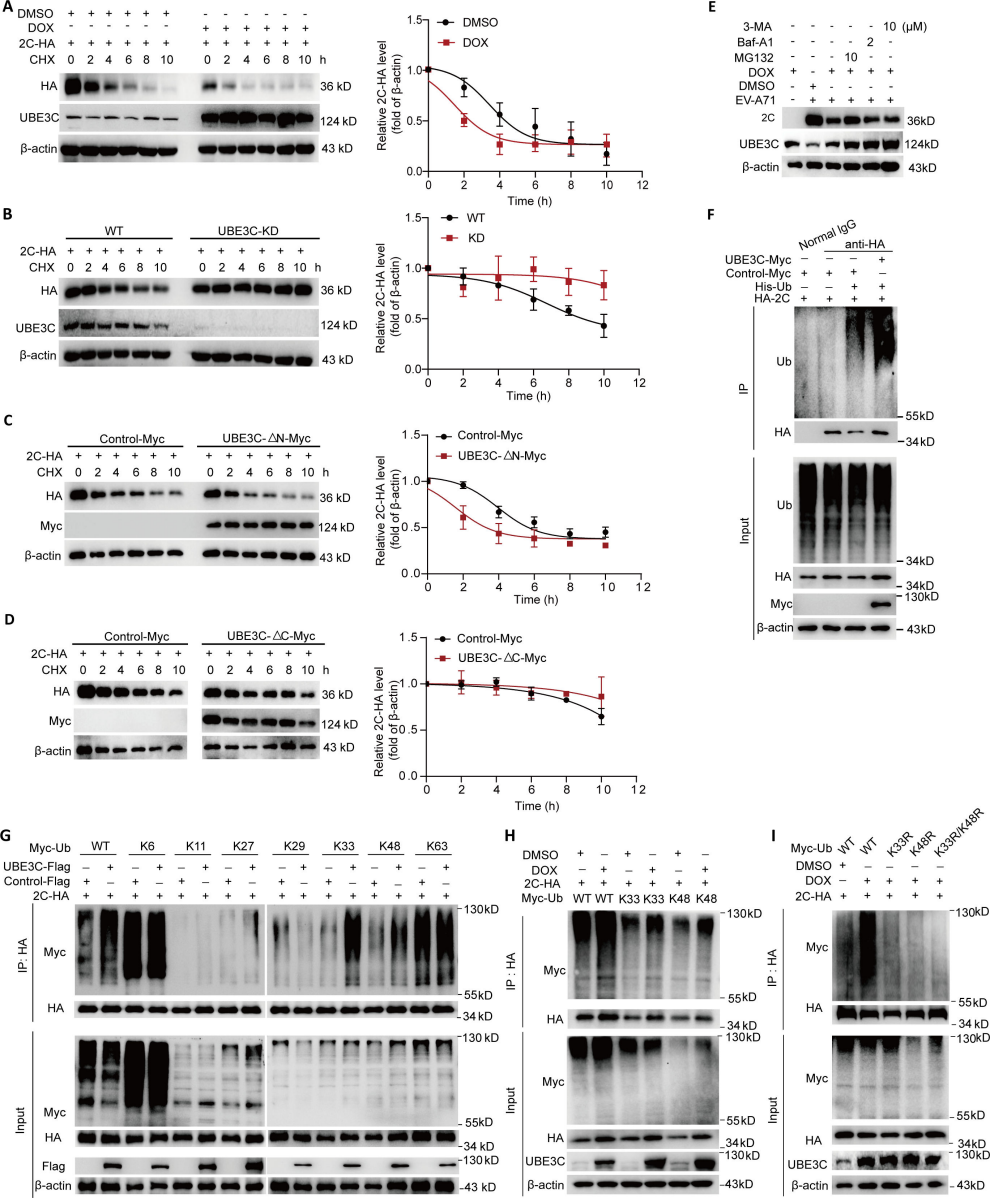
UBE3C facilitates K33/K48-linked ubiquitination of 2C for degradation. (**A**) The UBE3C-HEK293T cell line supporting DOX-induced UBE3C expression was treated with DOX or DMSO, and then cells were transfected with a plasmid expressing 2C-HA for 24 h. The cells were treated with 20 µg/mL of CHX and harvested at the indicated times for the western blot assay. Relative 2C-HA protein levels were quantified, and the mean values and SD of two replicates were determined in two independent experiments. (**B**) UBE3C-KD HEK293T cells and wide-type HEK293T cells were transfected with a plasmid-expressing 2C-HA for 24 h. The cells were treated with 20 µg/mL of CHX and harvested at the indicated times for the western blot assay. Relative 2C-HA protein levels were quantified and the mean values and SD of two replicates were determined in two independent experiments. (**C, D**) HEK293T cells were transfected with the indicated plasmids for 24 h. The cells were treated with 20 µg/mL of CHX and harvested at the indicated times for the western blot assay. Relative 2C-HA protein levels were quantified, and the mean values and SD of three replicates were determined in three independent experiments. (**E**) The HEK293T cell line supporting DOX-induced UBE3C expression was treated with DOX or DMSO, and then cells were infected with EV-A71 (H, MOI = 0.1) for 16 h. Then, cells were treated with DMSO, MG132, Baf-A1, or 3-MA for 8 h. The cells were harvested for western blot assay with the indicated antibodies. (**F, G**) HEK293T cells were transfected with the indicated plasmids for 24 h and treated with MG132 for 6 h. The cell lysates were subjected to immunoprecipitation with an HA-tagged mouse monoclonal antibody or normal IgG. The precipitated proteins were resolved by SDS-PAGE and probed with the indicated specific antibodies. (**H, I**) HEK293T cell line supporting DOX-induced UBE3C expression was treated with DOX or DMSO, and then cells were transfected with the indicated plasmids for 24 h and treated with MG132 for 6 h. The cell lysates were subjected to immunoprecipitation with HA tag mouse monoclonal antibody. The precipitated proteins were resolved by SDS-PAGE and probed with an HA-tagged indicated specific antibodies.

As an E3 ubiquitin ligase, UBE3C has been reported to be involved in a variety of ubiquitination modification processes, so it is conceivable that the binding of UBE3C to 2C may affect the stability of 2C protein. To test this hypothesis, we investigate the effect of UBE3C on the stability of 2C protein. The UBE3C-HEK293T cell line supporting DOX-induced UBE3C expression was treated with DOX or dimethyl sulfoxide (DMSO), and then cells were transfected with a plasmid-expressing 2C-HA for 24 h. The cells were treated with 20 µg/mL of CHX and harvested at the indicated times for the western blot assay to quantify the relative 2C-HA protein levels. As shown in Fig. 4A, the degradation rate of 2C protein in cells with inducible UBE3C expression was significantly increased compared with DMSO treatment. The degradation rate of 2C protein was significantly decreased in UBE3C knockdown cells (Fig. 4B), thus supporting the above results. Moreover, consistent with the interaction between UBE3C and 2C through its C-terminal, UBE3C-ΔN-Myc can promote the degradation of 2C (Fig. 4C), while UBE3C-ΔC-Myc has no significant effect on the stability of 2C (Fig. 4D). Autophagy and the ubiquitin-proteasome system are two classical ways of regulating protein degradation. To preliminarily determine the pathway of 2C degradation regulated by UBE3C, we treated cells with proteasome inhibitor MG132 and autophagy inhibitors Bafilomycin A1 (Baf-A1) and 3-Methyladenine (3-MA) to study their effects on 2C degradation regulated by UBE3C. The results showed that MG132 treatment can reverse the degradation of 2C by overexpressing UBE3C, while Baf-A1 and 3-MA have no effect on the degradation of 2C (Fig. 4E), indicating that UB3C regulates the degradation of 2C by the ubiquitin proteasome pathway. As expected, Co-IP analysis showed that EV-A71 2C was modified by ubiquitin, and UBE3C overexpression promotes 2C ubiquitylation (Fig. 4F). The process of ubiquitination degradation is varied and can occur at any one of the seven lysine residues of ubiquitin, resulting in the production of several ubiquitin chains that ultimately determine what happens to the altered protein. We found that UBE3C inhibited 2C ubiquitination by K33/K48 linkages in co-immunoprecipitation assay (Fig. 4G). This promotion of the K33/K48 ubiquitination modification of the 2C protein was observed in cells with stable expression of UBE3C (Fig. 4H). Moreover, the ability of UBE3C to regulate 2C ubiquitination was significantly deprived after K33R mutation, K48R mutation, and K33R/K48R double mutations of ubiquitin (Fig. 4I). In summary, our results provide evidence that UBE3C promotes the process of ubiquitination on K48 and K33 residues, leading to the degradation of 2C proteins and ultimately inhibiting the replication of EV-A71.

### K268 is the major ubiquitination site in 2C

**Fig 5:**
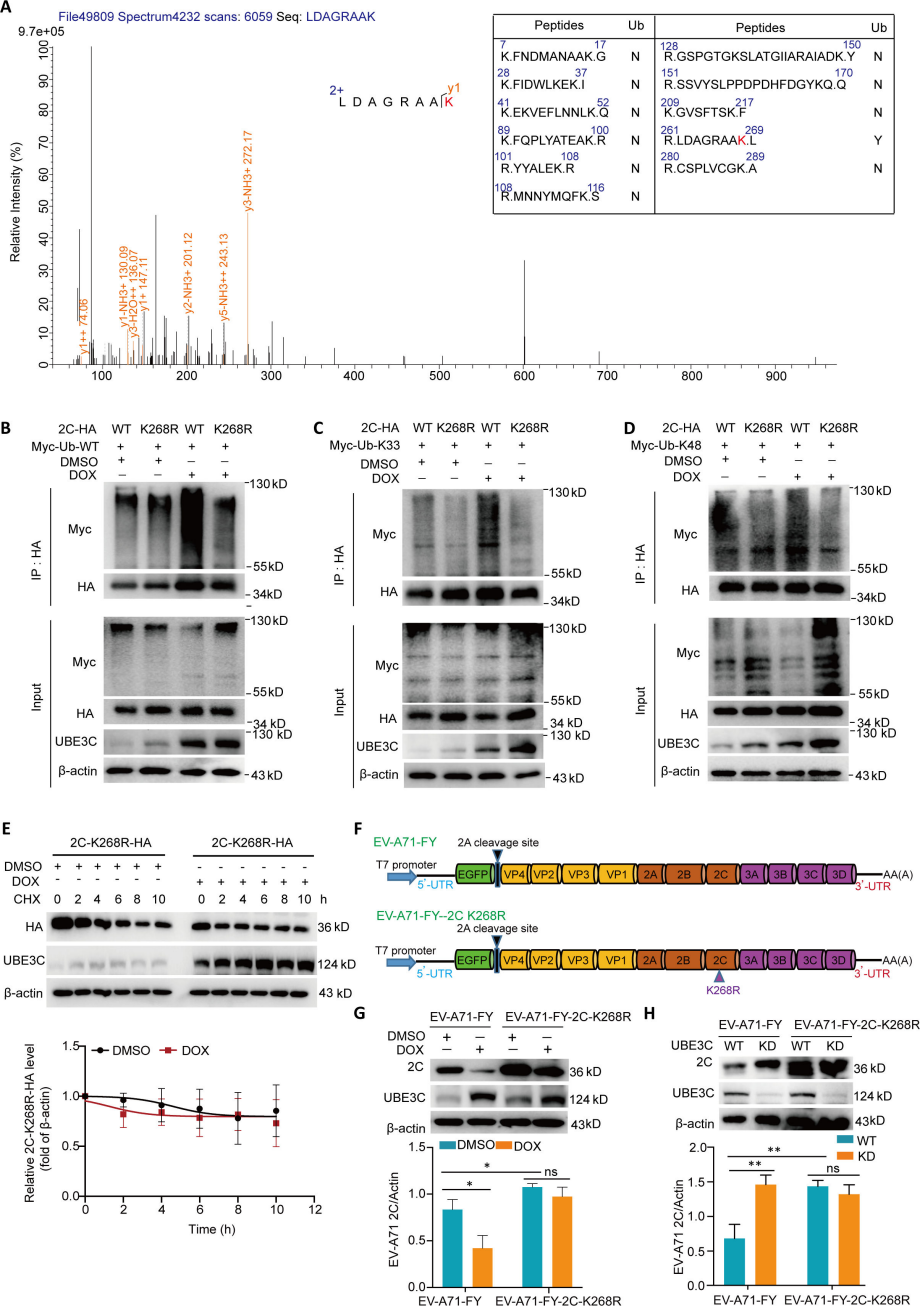
K268 is the major ubiquitination site in 2C (**A**) The abundance of 2C ubiquitination sites was identified by LC-MS/MS on 2C ubiquitinated in the presence or absence of UBE3C. Peak intensities are shown. (**B-E**) The HEK293T cell line supporting DOX-induced UBE3C expression was treated with DOX or DMSO, and then cells were transfected with the indicated plasmids for 24 h. The cell lysates were subjected to immunoprecipitation with an HA-tagged mouse monoclonal antibody. The precipitated proteins were resolved by SDS-PAGE and probed with the indicated specific antibodies. (**E**) The HEK293T cell line supporting DOX-induced UBE3C expression was treated with DOX or DMSO, and then cells were transfected with a plasmid-expressing 2C-K268R-HA for 24 h. The cells were treated with 20 µg/mL of CHX and harvested at the indicated times for the western blot assay. Relative 2C-K268R-HA protein levels were quantified, and the mean values and SD of two replicates were determined in two independent experiments. (**F**) Schematic map of the replication-competent EV-A71-FY and EV-A71-FY-2C K268R reporter viral genomes. (**G**) The UBE3C-HEK293T cell line supporting DOX-induced UBE3C expression was treated with DOX or DMSO, and then cells were infected with EV-A71-FY (MOI = 0.1) and EV-A71-FY-2C K268R (MOI = 0.1) for 36 h. The cells were harvested for western blot assay with the indicated antibodies (*n* = 3). (**H**) UBE3C-KD HEK293T cells and wide-type HEK293T cells were infected with EV-A71-FY (MOI = 0.1) and EV-A71-FY-2C K268R (MOI = 0.1) for 36 h. The cells were harvested for western blot assay with the indicated antibodies (*n* = 3). *P* < 0.05, student’s *t*-test.

To determine the ubiquitination modification site of the 2C protein induced by UBE3C, we utilized Co-IP combined with ubiquitination modification mass spectrometry. The mass spectrometry data indicated that amino acid 268 of 2C underwent ubiquitination modification site change (Fig. 5A). To confirm this finding, we created a 2C-HA mutant by replacing the lysine residue at position 268 with arginine. The results showed that Myc-Ub, Myc-Ub-k33, and Myc-Ub-k48 all promoted the ubiquitination of 2C-HA under UBE3C overexpression, while Myc-Ub, Myc-Ub-k33, and Myc-Ub-k48 did not affect the ubiquitination of 2C-HA K268R, suggesting that K268 is the main ubiquitination modification site for 2C (Fig. 5B through D). Moreover, the degradation rate of 2C-K268R-HA protein was not increased in cells that induced UBE3C expression (Fig. 5E). Subsequently, to gain a deeper understanding of how 2C K268R ubiquitination modification affects EV-A71 infection, cDNA encoding a EV-A71-FY with 2C K268R mutation (EV-A71-FY-2C K268R) was obtained by point mutation on our previously constructed EV-A71-FY clone (Fig. 5F) (12). The results showed that 2C K268R mutation increased the levels of 2C protein in the infected wide-type cells or infected UBE3C-HEK293T cells without DOX treatment as revealed by western blot assay (Fig. 5G and H). As anticipated, the replication of EV-A71-FY-2C-K268R was not significantly affected by the elevated or decreased UBE3C expression level (Fig. 5G and H). These results indicate that 2C K268 is the main ubiquitination site regulated by UBE3C.

### UBE3C also restricts the replication of CVB3 and CVA16 by promoting the ubiquitination of 2C

**Fig 6:**
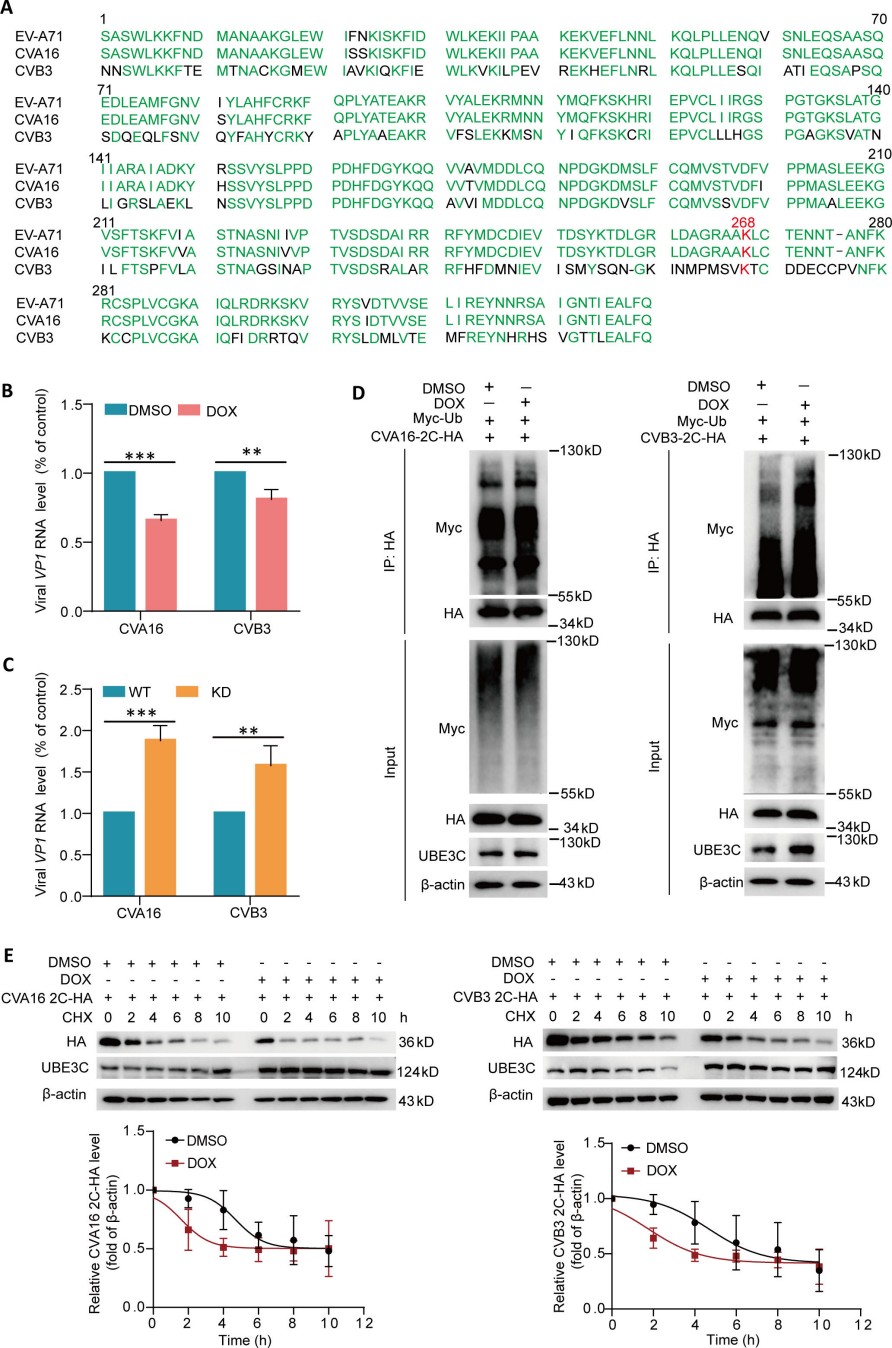
UBE3C also restricts the replication of CVB3 and CVA16 *via* promoting the ubiquitination of 2C (**A**) Amino acid sequence alignment analysis of 2C proteins of EV-A71, CVB3, and CVA16. (**B**) HEK293T cell line supporting DOX-induced UBE3C expression was treated with DOX or DMSO, and then cells were infected with CVA16 or CVB3 (MOI = 0.1) for 24 h. The cells were harvested for the qRT-PCR assay (*n* = 3). (**C**) UBE3C-KD HEK293T cells and wide-type HEK293T cells were infected with CVA16 or CVB3 (MOI = 0.1) for 24 h. The cells were harvested for the qRT-PCR assay (*n* = 3). (**D**) HEK293T cells were transfected with the indicated plasmids for 24 h. The cell lysates were subjected to immunoprecipitation with HA-tagged mouse monoclonal antibody. The precipitated proteins were resolved by SDS-PAGE and probed with the indicated specific antibodies. (**E**) The UBE3C-HEK293T cell line supporting DOX-induced UBE3C expression was treated with DOX or DMSO, and then cells were transfected with a plasmid expressing CVA16 2C-HA or CVB3 2C-HA for 24 h. The cells were treated with 20 µg/mL of CHX and harvested at the indicated times for the western blot assay. Relative CVA16 2C-HA or CVB3 2C-HA protein levels were quantified, and the mean values and SD of three replicates were determined in two independent experiments.

Consistent with literature reports, through amino acid sequence comparison analysis, we found that the 2C amino acid sequence of EV-A71 (BrCr, GenBank accession number AAB39968), CVB3 (GenBank accession number AAA42931), and CVA16 (GenBank accession number AUF49660) was highly conserved, and the 268 position of the three viruses was all lysine, suggesting that UBE3C may have the same inhibitory effect on CVB3 and CVA16 (Fig. 6A). To test this hypothesis, we evaluated the effects of UBE3C on the replication of CVB3 and CVA16 in induced UBE3C expression or UBE3C knockdown cells, respectively. As shown in Fig. 6B and C, the induced expression of UBE3C significantly decreased the RNA levels of intracellular CVB3 VP1 and CVA16 VP1, while UBE3C knockdown significantly increased the RNA levels of intracellular CVB3 VP1 and CVA16 VP1. As shown in Fig. 6D, co-immunoprecipitation analysis showed that CVB3 2C and CVA16 2C were both modified by ubiquitin, and UBE3C overexpression promotes CVB3 2C and CVA16 2C ubiquitylation. Moreover, the degradation rates of CVA16 2C and CVB3 2C proteins in cells induced to express UBE3C were significantly increased (Fig. 6E). Taken together, our results suggest that UBE3C also plays a limiting role in the replication of CVB3 and CVA16.

## DISCUSSION

Post-translational modifications (PTMs) are crucial for preserving cellular functionality. Specialized enzymes, such as ubiquitin E3 ligase, glycosyltransferase, poly ADP (adenosine diphosphate) ribose polymerase (PARP), acetyltransferase, and kinases, carry out these modifications to enhance protein solubility, interactions, and degradation (21). These modifications can have an antiviral effect by stimulating an immune response or targeting viral protein degradation. For instance, ZIKV and yellow fever viruses attach to RIG-I and block its ubiquitination, which, in turn, inhibits the phosphorylation and nuclear translocation of IRF3, thereby inhibiting the IFN response (21, 22).

PTMs of various viral proteins have been found during enteroviruses infections. Ubc 9 interacts with the EV-A71 3C protein to promote SUMO modification of the 3C protein to help host cells defend against viral replication and induce apoptosis (23). A host E3 ubiquitin ligase speckle-type POZ protein (SPOP) increases lysosomal-dependent degradation of EV-A71 2A protein by activating K48-linked polyubiquitination of 2A protein and restricting the replication of EV-A71 (13). The m6A methyltransferase METTL3 interacts with EV-A71 RNA-dependent RNA polymerase, causing SUMOylation and ubiquitination to enhance its stability (24). E3 ligase XIAP is crucial for the neddylation of the EV-A71 VP2 protein, which results in its degradation and hinders EV-A71 development (12). ISGylation of the CVB3 2A protein inhibits eIF4G1 cleavage and viral propagation (25). In short, PTMs of viral proteins to degrade or regulate their function are one of the strategies of the host to resist viral infection.

Enterovirus 2C protein is highly conserved and has multiple functions, and it has great potential as a target for enterovirus therapy. To facilitate viral RNA replication, the 2C protein performs the function of an RNA helicase as well as an ATP-independent chaperone action (26). Studies reveal that TRIM7 affects viral RNA replication *via* regulating 2BC protein (27). The post-translational modification of the 2C protein may affect its function. However, there are few studies on the post-translational modification of 2C protein. In this study, we confirmed the interaction between human E3 ligase UBE3C and EV-A71 2C for the first time by CO-IP combined with LC-MS/MS analysis and other methods (Fig. 1). We also found that the C-terminal of UBE3C is the main region of interaction with 2C, and this interaction is independent of its enzyme active site K903 (Fig. 2). In addition, this interaction promotes the ubiquitination and degradation of 2C, hence raising the host cell’s capacity to resist EV-A71 invasion (Fig. 3 and 4). It is interesting to note that although the antiviral effect of UBE3C-ΔC is weaker than that of full-length UBE3C, the deletion of the C-terminal domain of UBE3C still has an antiviral effect (Fig. 3N). This corresponds to the interaction between the C-terminal domain of UBE3C and 2C, but it also suggests that there may be other mechanisms other than the regulation of 2C by UBE3C to inhibit viral replication. More importantly, we found that UBE3C also promotes ubiquitination of the 2C proteins of CVB3 and CVA16, thereby limiting viral replication (Fig. 6).

UBE3C has been reported to promote various types of ubiquitination, but it has not been reported during viral infection. Studies show that UBE3C could regulate autophagy through K33-linked ubiquitination while preventing ATG4B degradation (17). IL-13RA2 acted as a modular link to enhance the contact between UBE3C and p53, resulting in the induction of K48-linked ubiquitination of p53 (28). It was found that UBE3C regulated the K29/K48 ubiquitination of the class III PI3-kinase complex (also known as the VPS34 complex), thereby enhancing the binding of VPS34 to the proteasome and inhibiting the formation and maturation of the autophagosome (10). In this study, we demonstrated for the first time that the EV-A71 2C protein underwent K33/K48 ubiquitination modification, leading to protein degradation (Fig. 4). However, we only confirmed the existence of K33 and K48 ubiquitination types, their exact combination mode is unknown. Moreover, our results showed that the K33R or K48R single mutation of ubiquitin had the same effect on the ubiquitination of UBE3C-regulated 2C as the K33R/K48R double mutations (Fig. 4I), indicating that the 2C protein may undergo multiple ubiquitination modifications of K33/K48, which needs more research to confirm in the future. Interestingly, studies have demonstrated that SERINC 5 undergoes polyubiquitination at the amino acid residue K130 through K33 and K48 linked ubiquitin chains. Polyubiquitylation via K33 linkages dictates the expression of SERINC 5 at the plasma membrane, while polyubiquitylation *via* K48 linkages leads to the downregulation of SERINC 5 at the cell surface (11). This suggests that UBE3C may also affect the localization and enzyme activity of 2C proteins, which is what we need to further investigate in the future, including more precisely confirming the presence of K33 and K48 forms.

Through LC-MS combined with mutagenesis and biochemical analysis, the site of 2C ubiquitination was mapped to residue K268. Substitution of K268 with arginine (R) completely eliminated ubiquitination of 2C regulated by UBE3C, and overexpression of UBE3C did not reduce the protein stability of 2C-K268R-HA. (Fig. 5). More importantly, UBE3C knockdown had no effect on the level of the EV-A71-FY-2C K268R 2C protein (Fig. 5). The 2C protein belongs to the SF-3 helicase family and is structurally composed of four subdomains, including the N-terminal membrane binding, ATPase, zinc finger, and C-terminal domain (29). The cysteine-rich zinc finger motif is situated between positions 270 and 286. Multiple residues and sites can affect 2C function by affecting the zinc finger domain. For example, C270, C281, and C286 are essential for the EV-A71 2C protein to fold appropriately (5, 26). The position of K268 is immediately adjacent to the zinc finger domain, and whether the binding of UBE3C to 2C will affect the normal folding of 2C protein and the binding of 2C to other host proteins needs further experiments to prove.

**Fig 7:**
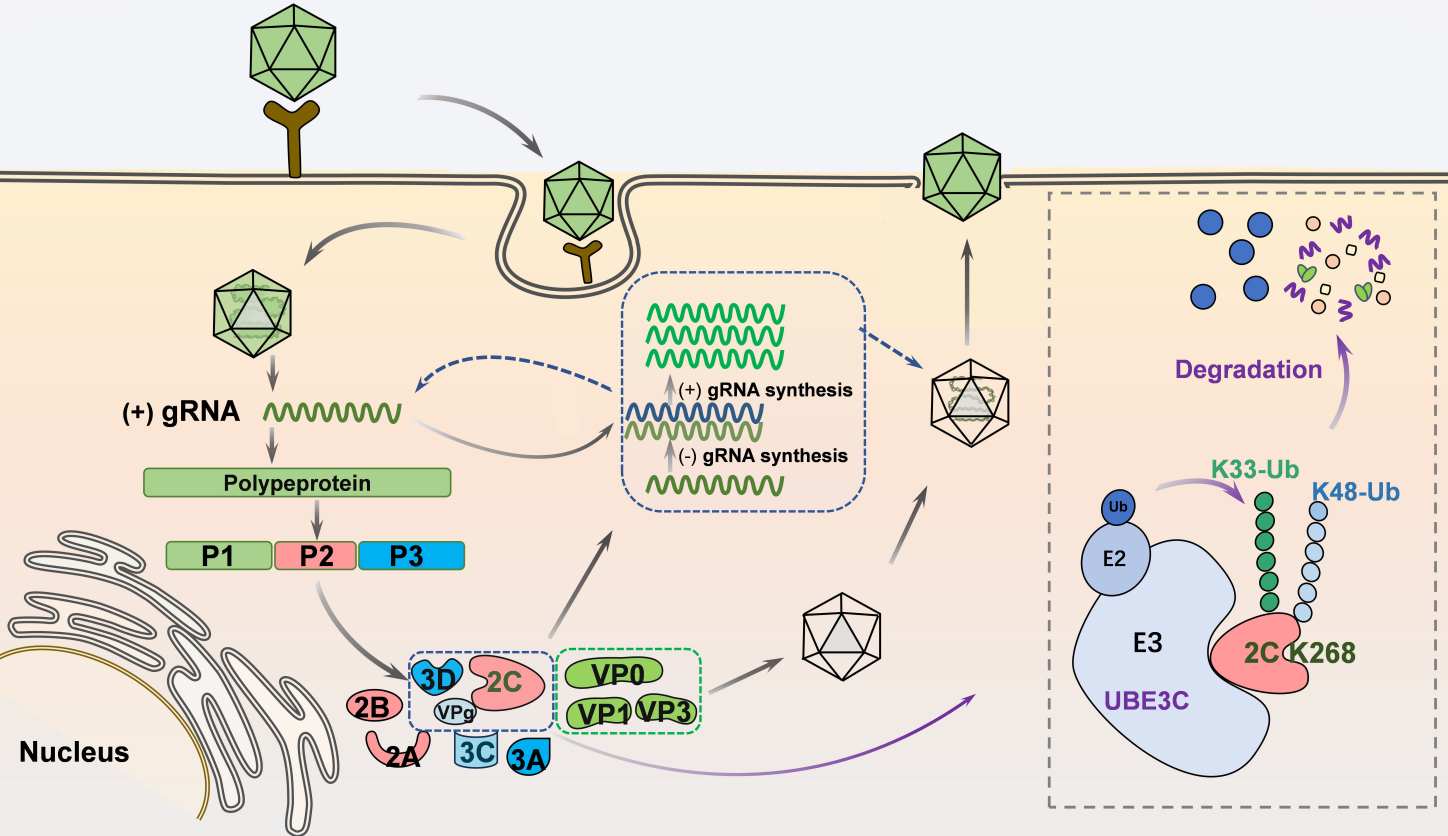
A model for UBE3C-mediated inhibition of EV-A71 replication. EV-A71 virus encodes non-structural protein for transcription to produce virus particles. Host ubiquitin E3 ligase UBE3C interacts with 2C and induces K33/K48-linked polyubiquitination of 2C K268, which results in the degradation of 2C. UBE3C-mediated degradation of 2C ultimately inhibits EV-A71 replication.

In summary, we determined for the first time that UBE3C regulates the K33/K48 ubiquitination modification at K268 of the EV-A71 2C protein (Fig. 7). Our discovery of UBE3C-mediated 2C ubiquitination may provide new insights into the control of enterovirus replication, including EV-A71, and provide new targets for enterovirus therapy.

## MATERIALS AND METHODS

### Cells and viruses

African green monkey kidney (Vero) cells were purchased from the American Type Culture Collection (ATCC) and cultured in modified Eagle’s medium (Invitrogen, Carlsbad, CA, USA) in Dulbecco’s modified Eagle medium (Invitrogen, Carlsbad, CA, USA) supplemented with 10% fetal bovine serum (FBS) and antibiotics (100 U/mL penicillin and 100 mg/mL streptomycin) at 37°C in a 5% CO_2_ incubator. Human embryonic kidney (HEK293T) cells and human colon cancer (HCT-8) cells were purchased from the Cell Culture Center of Peking Union Medical College or Chinese Academy of Sciences and cultured in Dulbecco’s modified Eagle medium (Invitrogen, Carlsbad, CA, USA) supplemented with 10% FBS and antibiotics (100 U/mL penicillin and 100 mg/mL streptomycin) at 37°C in a 5% CO_2_ incubator. UBE3C-Knockdown (UBE3C-KD) HEK293T cells (L11057) were purchased from Beyotime Biotechnology (Shanghai, China).

EV-A71 strain H (VR-1432) and strain BrCr (VR-1775) and CVB3 strain Nancy were all purchased from ATCC. EV-A71 strain SZ98 and CVA16 strain shzh05-1/GD/CHN/2005 were kindly provided by Dr. Jianwei Wang, Institute of Pathogen Biology, Chinese Academy of Medical Science, and Peking Union Medical School. EV-A71 strain JS-52 was kindly provided by Dr. Xiangzhong Ye, Beijing Wantai Biological Pharmacy Enterprise Co., Ltd. All viruses were passaged in Vero cells.

### Generation of stable cell lines

To generate cells stably expressing UBE3C, lentivirus pCV051 UBE3C (DOX-on) and lentivirus pCV051 vector (DOX-on) were purchased from Shanghai Genechem. Co. (Shanghai, China) and were then infected into HEK293T or HCT-8 cells. After 16  h of infection, stable cells were selected in a medium containing 2 µg/mL or 10 µg/mL puromycin (Gibco, CA, USA). The cultured cloned cells were used for follow-up experiments. The stable expression of UBE3C was induced by adding 2 µg/mL DOX to the cells.

### Production of EV-A71-FY-2C K268R reporter viruses

The cDNA encoding an EGFP-EV-A71 with 2C K268R mutation (EV-A71-FY-2C K268R) was obtained by a point mutation on our previously constructed EV-A71-FY clone (12). EV-A71-FY and EV-A71-FY-2C K268R RNAs were prepared through *in vitro* transcription with a Not-I-linearized plasmid harboring EV-A71-FY and EV-A71-FY-2C K268R as a template. An mMESSAGE mMACHINE T7 transcription kit (Invitrogen) was used. Vero cells were transfected with EV-A71-FY and EV-A71-FY-2C K268R RNA with Lipofectamine 3000 (Invitrogen). EV-A71-FY and EV-A71-FY-2C K268R viruses were collected by three freeze-thaw cycles and frozen at −80°C until use.

### Compounds

Cycloheximide (CHX) was purchased from Sigma-Aldrich (St. Louis, MO, USA). MG132, Bafilomycin A1, 3-Methyladenine, and Doxycycline were purchased from MedChemExpress (Monmouth Junction, NJ, USA).

### Plasmids

Plasmids expressing UBE3C-Myc, Myc-tagged negative control vector (Control-Myc), Flag-tagged negative control vector (Control-Flag), and pCMV3-C terminally HA-tagged negative control vector (Control-HA) were purchased from Sino Biological Inc. (Beijing, China). Plasmids expressing EV-A71-2C-HA, CVA16-2C-HA, CVB3-2C-HA, EV-71–2C-HA (K268R), UBE3C-K903R-Flag, UBE3C-K903R-Myc, UBE3C-delete-C terminal, and UBE3C-delete-N terminal were purchased from Inovogen Tech. Co. (Chongqing, China). Plasmids expressing ITCH-Flag, UBE3C-Flag, ZNF598-Flag, UHRF1-Flag, WWP2-Flag, MKRN2-Flag, Myc-STUB1, UBR5-Myc, MARCHF5-Flag, Ub-Myc, Ub-K6-3xMyc, Ub-K11-Myc, Ub-K27-Myc, Ub-K29-Myc, Ub-K33-Myc, Ub-K48-Myc, and Ub-K63-Myc were purchased from miaoling Biology (Wuhan, China).

### Immunoprecipitation

HEK293T cells were transfected with the indicated plasmids using Lipofectamine 3000 (Invitrogen) for 24 h. The cells were then lysed using Pierce IP lysis buffer (Thermo Fisher Scientific, Waltham, MA, USA) containing a halt protease inhibitor single-use cocktail (Thermo) for 30 minutes on ice. Lysates were incubated with HA-Nanoab-Agarose beads (HNA-250–5K, LABLEAD) overnight at 4°C or protein A agarose beads (Roche, Basel, Switzerland) at 4°C for 3 h. The beads were washed five times with cold PBS. Complexes were separated from the beads and then boiled with 2 × sample buffer (Thermo) for 10 minutes. The precipitated proteins were subjected to SDS-PAGE and probed with the indicated specific antibodies (30).

### Real-time qRT-PCR

Total RNA was isolated from cells using the RNeasy Mini Kit (Qiagen, Hilden, Germany) and analyzed with the TransScript II Green one-step qRT-PCR SuperMix (TransGen Biotech) using the ABI 7500 fast real-time PCR system (Applied Biosystems). The mRNA expression of CVB3 VP1 was detected with the sense primer 5′-ACATGGTGCGAAGAGTCTATTGAG-3′ and the antisense primer 5′-TGCTCCGCAGTTAGGATTAGC-3′, targeting a conserved region of the VP1 gene. The mRNA expression of CVA16 VP1 was detected with the sense primer 5′-GTTATCCCACCTTCGGAGA-3′ and the antisense primer 5′-TCGGGCATTGACCATAATCTAG-3′, targeting a conserved region of the VP1 gene. The GAPDH mRNA was detected using the sense primer 5′-GAAGGTGAAGGTCGGAGTC-3′ and the antisense primer 5′-GAAGATGGTGATGGGATTTC −3′.

### Western blot

Whole-cell lysate was extracted by M-PER mammalian protein extraction reagent (Thermo) containing Halt protease inhibitor single-use cocktail (Thermo). Protein extracts were separated by SDS-PAGE and transferred onto polyvinylidene difluoride (PVDF) membranes. The transferred membranes were blocked by 5% skim milk for 60 min at room temperature and then incubated with the indicated primary antibodies at 4°C for 12 h, followed by incubation with the corresponding secondary antibodies at room temperature for 1 h. The primary antibodies used in this study included antibodies against β-actin (1:1,000 dilution; Cell Signaling Technology, Beverly, MA, USA), UBE3C (1:1,000 dilution; GeneTex), EV-A71 2C (1:1,000 dilution; GeneTex, CA, USA), EV-A71 VP1 (1:1,000 dilution; GeneTex, CA, USA), EV-A71-3AB (1:1,000 dilution; GeneTex), EV-A71-2B (1:1,000 dilution; GeneTex), and EV-A71-3CD (1:1,000 dilution; GeneTex), Flag rabbit monoclonal antibody (1:1,000 dilution; Cell Signaling Technology), HA rabbit monoclonal antibody (1:1,000 dilution; Cell Signaling Technology), and Myc rabbit monoclonal antibody (1:1,000 dilution, Cell Signaling Technology) (31).

### LC-MS/MS assay

Pierce IP lysis buffer (Thermo Fisher Scientific, Waltham, MA, USA) was used to extract the cells transfected with 2C-HA. The whole lysates were incubated with anti-HA agarose at 4°C for 12 h. Then, the IP complexes were obtained by centrifugation at 2,500 × *g* for 2 min and washed five times. After that, the protein samples were subjected to SDS-PAGE, and Coommassie Brilliant Blue staining (CBB) was performed to visualize differential proteins that were delivered to LC-MS/MS analysis (Bgi Genomics Co., China) to identify the interacting proteins and arginine modification site.

### Surface plasmon resonance

SPR analysis was performed on a CM5 SPR chip integrated into the Reichert4 SPR system (Reichert, Buffalo, NY, USA). All solutions were prepared using ultrapure water obtained from the Master Touch-S15UVF Pure Water Purification System. The running buffer used throughout the analysis was PBST (pH 7.6 PBS buffer, 0.05% Tween-20), which was filtered through a 0.22-µm membrane filter before use. The UBE3C protein (TP315110, ORIGENE) was prepared with pH 4.5 sodium acetate solution at 200 µL, 0.25 µg/µL. The analysis temperature was controlled at 25±1°C. The target/control proteins were immobilized on the surface of the gold film of the chip containing carboxymethyl glucan by covalent amine coupling. The CM5 SPR chip is composed of four channels, the fourth channel is set as the reference channel, and the other three channels are the sensing channels. The UBE3C proteins were immobilized on the sensing channel, and the reference channel was activated but not immobilized. Furthermore, ethanolamine was used as a blocker to fill in the blanks. The final signal from the sensing channel is normalized by subtracting the signal from the reference channel. Target and control proteins were injected into the respective sensing channels at a rate of 10 µL/min for 300 s, and the CM5 chip was ready for the immobilization of proteins. Then 2C protein from the concentration gradient was successively injected into the SPR channels at a speed of 25 µL/min to collect the real-time SPR signal.

### Titration of virus yield

The cells were harvested at the indicated times after the EV-A71 infection. After three freeze-thaw cycles of cells and culture medium, the titers of the virus in the cell lysates were determined in Vero cells by a cytopathic effect assay. The method of Reed and Muench was used to determine 50% tissue culture infective doses (TCID_50_) (32).

### Quantification and statistical analysis

Statistical analyses were performed using the GraphPad Prism 9.0 software. Results are expressed as mean ± SD. Statistical significance was assessed using an unpaired two-tailed Student’s *t*-test or one-way ANOVA with the Holm-Sidak multiple comparisons test. *P* < 0.05 was considered significant.

## Data Availability

The data associated with this paper are available upon request to the corresponding author.
